# Thermal-Responsive Antibacterial Hydrogel with Photothermal Therapy and Improving Wound Microenvironment for Promote Healing

**DOI:** 10.3390/antiox13070857

**Published:** 2024-07-17

**Authors:** Linjie Huang, Jingwen Deng, Yina Su, Xueqi Hu, Yichao Zhang, Shanni Hong, Xiahui Lin

**Affiliations:** 1 School of Medical Imaging, Fujian Medical University, Fuzhou 350122, China; linjie_huang19@163.com (L.H.); suyina0525@163.com (Y.S.); hxq1196735422@163.com (X.H.); 18965527139@163.com (Y.Z.); 2 Department of Otorhinolaryngology, Fujian Medical University Union Hospital, Fuzhou 350001, China; libwendy@163.com

**Keywords:** skin damage, bacterial infection, wound microenvironment, photothermal therapy, oxidative stress

## Abstract

Skin damage is one of the most prevalent human injuries, which affects the health of human beings. However, skin damage is often accompanied by bacterial infection and wound microenvironment changes, causing damage to normal cells and inhibiting wound healing. Herein, we designed a thermal-responsive antibacterial hydrogel (GAG hydrogel) loaded with catalase (CAT)-like Au@Pt@MgSiO_3_ nanoparticles (APM NPs) and gentamicin (GM) to promote wound healing. The GAG hydrogel was used in a photothermal therapy (PTT)/antibiotic combination to kill bacteria, reduce the use of antibiotics, improve the wound microenvironment, promote cell proliferation, and accelerate wound healing. Under near-infrared laser irradiation, APM NPs in the hydrogel generated local hyperthermia to kill bacteria. Meanwhile, the generated heat led to a change in the hydrogel’s morphology, enabling it to release GM and APM NPs to prevent the overuse of antibiotics. Subsequently, the CAT-like ability of the APM NPs decreased the oxidative stress caused by hydrogen peroxide (H_2_O_2_), thus remodeling the wound microenvironment. Then, the weakly acidic microenvironment of the wound caused the decomposition of the APM NPs and the release of magnesium ions (Mg^2+^), promoting the growth and migration of cells for wound healing. Therefore, the studied thermal-responsive antibacterial (GAG) hydrogel has potential in the field of wound healing.

## 1. Introduction

The skin, as the largest organ in the human body, covers the entire body’s surface and serves as a natural barrier. It plays a vital role in maintaining body fluids and preventing the invasion of harmful substances and pathogenic microorganisms [1]. As a prevalent human injury, skin damage is often accompanied by bacterial infection, oxidative stress, inflammation, and cell proliferation disorders [2,3,4]. Once bacteria accumulate, persistent chronic inflammation often occurs in the wound area, which hinders damaged tissues’ transition and further delays wound healing [5]. Furthermore, bacterial infection can cause changes in the wound microenvironment, including oxidative stress and a weakly acidic microenvironment [6]. These changes may hinder cell proliferation and slow down wound healing [7,8,9,10,11]. Thus, designing a new strategy that can enable the provision of antibacterial treatment and regulation of the wound microenvironment is of great significance for accelerating wound healing. In the past few decades, antibiotic therapy has been widely used in antibacterial therapy because of its high efficiency and universality, but the excessive use and abuse of antibiotics has led to the emergence of drug-resistant strains, which considerably affect the therapeutic effect of antibiotics [12,13]. Methicillin-resistant *Staphylococcus aureus* (MRSA) is a common and highly toxic drug-resistant bacterium that is difficult to kill with antibiotics [14]. When a wound is infected with MRSA, traditional doses of antibiotics are insufficient to effectively kill bacteria, impeding tissue regeneration and delaying wound healing. In addition, due to the absence of skin on the wound, the continued presence of bacteria further exacerbates the deterioration of the wound microenvironment and causes damage to normal cells, making it difficult for the wound to heal [15]. Therefore, a multifunctional strategy must be designed that can effectively inhibit bacterial infection and optimize the microenvironment of wounds, which will play a crucial role in wound-healing treatment.

Photothermal therapy (PTT), as a novel non-invasive treatment strategy, has a unique advantage in inhibiting wound infection [16,17,18]. It has been reported that local hyperthermia generated by photothermal agents (PTAs) under laser irradiation could destroy the structure of bacteria [16,17,18]. With the development of nanotechnology, more and more PTA-based nano-antibacterial agents have been designed and used to promote wound healing, such as metal nanostructure-based nanoparticles, transition metal sulfide/oxide nanomaterials, and carbon-based PTAs [19]. Under laser irradiation, this type of nano-antibacterial agent can generate local hyperthermia to effectively kill bacteria. The degree of hyperthermia can be adjusted by changing the laser parameters, achieving controllable regulation of the antibacterial effect and reducing the use of antibiotics and unnecessary thermal damage [20]. Additionally, an appropriate heating temperature (below 45 °C) improves the permeability of bacterial cell membranes and accelerates the penetration of antibiotics, as well as rendering previously resistant bacteria sensitive to antibiotics, which has proven useful in the new antibacterial treatment strategy of PTT combined with antibiotics [21]. Therefore, the combination of PTT and low-dose antibiotics can effectively prevent the overuse of antibiotics and has the potential to be applied in wound-healing treatments.

Wound microenvironment change is a complex process caused by bacterial infection and inflammation, and it is mainly characterized by oxidative stress and the inhibition of fibroblast proliferation [22]. Oxidative stress caused by the overexpression of hydrogen peroxide (H_2_O_2_) damages normal cells at the wound site and inhibits wound healing [23]. Furthermore, as an important factor in tissue regeneration, fibroblast proliferation is inhibited by wound microenvironment changes, which is detrimental to wound healing [24]. Therefore, alleviating oxidative stress by reducing H_2_O_2_ and promoting fibroblast proliferation to modulate the wound microenvironment has a positive effect on wound healing. Some catalases (CAT) and their enzyme mimics (such as platinum nanoparticles) can catalyze the decomposition of H_2_O_2_ to produce oxygen (O_2_) and water, thereby reducing damage caused by oxidative stress [25,26]. However, due to the loss of skin tissue, the wound is repeatedly infected by bacteria, leading to a reduction in the effectiveness of CAT-related oxidative stress alleviation and deteriorating the wound microenvironment [15]. To ameliorate the wound microenvironment, wound shielding should be performed to reduce the continuous accumulation of external bacteria. Hydrogel-based wound dressings with good biocompatibility and adhesion are widely used for wound shielding and the treatment of various types of wounds [11,27]. Hydrogels can not only fill the gap left by lost skin to isolate external bacteria, but also simulate the extracellular matrix (ECM) to provide a suitable environment for cell proliferation and wound healing [28]. Gelatine methacrylate (GelMA), an ideal photo-crosslinked dressing, can be used to form hydrogels to simulate the ECM and fill in areas of missing skin [29,30]. GelMA hydrogel also has a good load capacity and a thermal-responsive capacity for drug loads, making it suitable for wound healing [31]. Moreover, upon doping with metal ions, hydrogels obtain additional functions [32]. For example, magnesium ions (Mg^2+^) in hydrogels can accelerate the migration and adhesion of cells, increasing the production of the ECM to promote wound healing [32,33]. Therefore, designing a thermal-responsive antibacterial hydrogel which can kill bacteria, reduce the use of antibiotics, and mediate the wound microenvironment is of great significance in the field of wound healing.

**Scheme 1:**
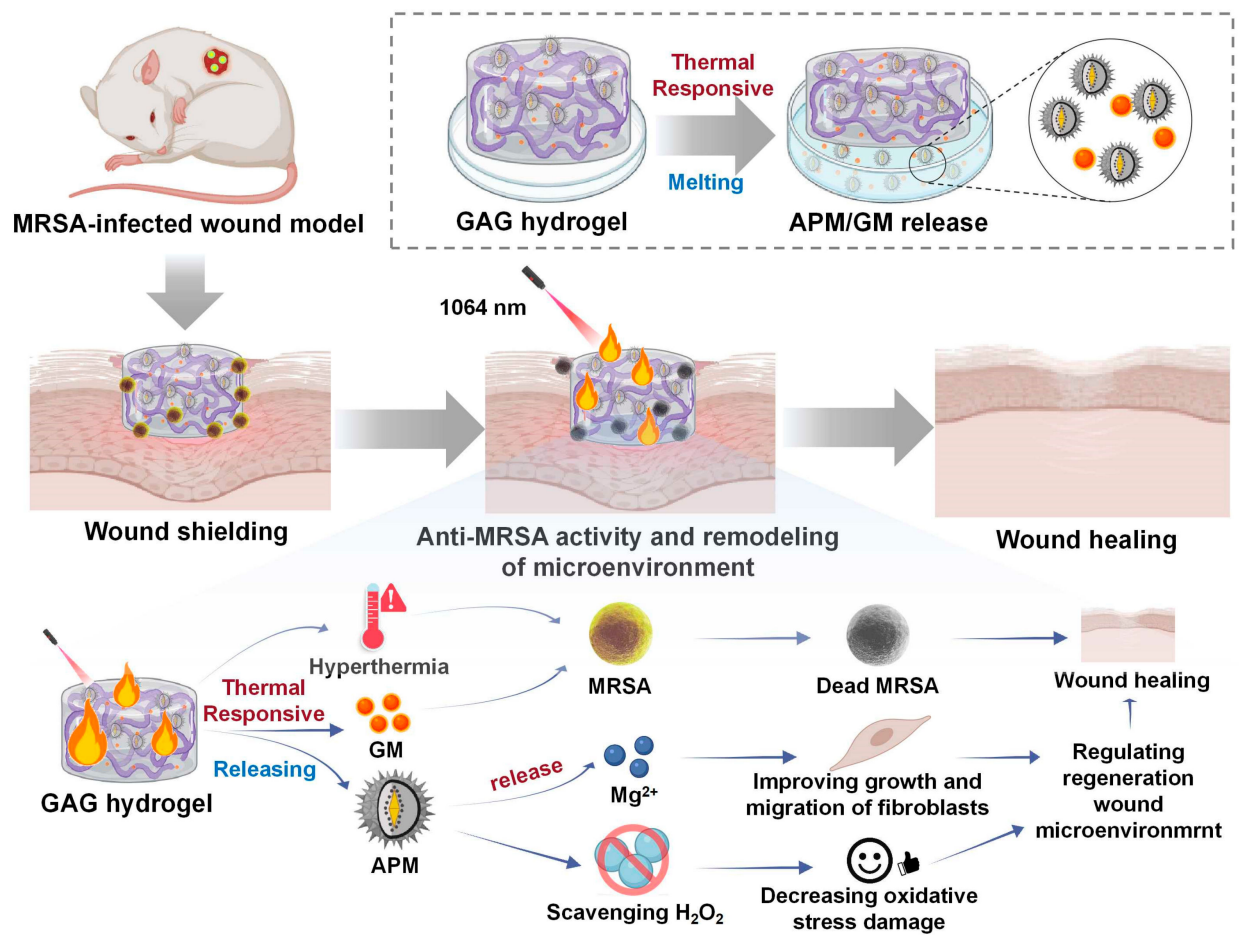
Illustration of thermal-responsive antibacterial GAG hydrogel for accelerating wound healing through a PTT/antibiotic combination to kill bacteria, reduce the use of antibiotics, remodel the wound microenvironment, and promote cell proliferation.

Herein, we designed a thermal-responsive GelMA hydrogel (GAG hydrogel) loaded with Au@Pt@MgSiO_3_ nanoparticles (APM NPs) as nano-antibacterial agents and gentamicin (GM) (Scheme 1). These consisted of gold–platinum bipyramid nanoparticles as the core and magnesium silicate (MgSiO_3_) as a shell, which provided them with photothermal conversion performance, H_2_O_2_ scavenging ability, and Mg^2+^ release capability. Under near-infrared (NIR) laser irradiation, the GAG hydrogel induced local hyperthermia to kill bacteria by relying on the internal APM NPs, and achieved the thermal-responsive release of APM NPs and GM to the wound area. The APM NPs distributed in the wound area effectively killed bacteria through PTT and reduced the dosage of antibiotics, and were able to further optimize the wound microenvironment. The CAT-like properties of APM NPs enabled the catalysis of H_2_O_2_ into O_2_ and water, reducing damage to the cells caused by oxidative stress. The acidic wound microenvironment induced the decomposition of APM NPs to generate Mg^2+^, which accelerated the growth and migration of fibroblasts, increasing ECM production and promoting wound healing. Therefore, due to its ability to kill bacteria and improve the wound microenvironment, this thermal-responsive antibacterial GAG hydrogel represents a new wound-healing strategy.

## 2. Materials and Methods

### 2.1. Materials

Gold chloride trihydrate (HAuCl_4_·3H_2_O), chloroplatinic acid hexahydrate (H_2_PtCl_6_·6H_2_O), hexadecyltrimethylammonium chloride (CTAC), sodium borohydride (AA), hexadecyltrimethylammonium bromide (CTAB), tetraethoxysilane (TEOS), ethanol, ammonia solution, silver nitrate (AgNO_3_), lithium phenyl(2,4,6–trimethyl benzoyl)phosphinate (LAP), and gelatin methacrylate were purchased from Aladdin (Wuhan, China). The MRSA strain was purchased from the American Type Culture Collection, Manassas, VA, USA. Luria–Bertani (LB) medium was obtained from Qingdao Hope Biotechnology (Qingdao, China). Mueller–Hinton broth (MHB) was obtained from Guangdong Huankai Microbial Sci. & Tech. SYTO 9/PI dye was acquired from Jiangsu Keygen Biotech (Nanjing, China). The L929 cell strain was purchased from the American Type Culture Collection (ATCC; Manassas, VA, USA). A cell counting kit-8 (CCK8) and a reactive oxygen species assay kit (DCFH–DA) were obtained from Beyotime (Shanghai, China). RPMI–1640 medium was obtained from Gibco (Billings, MT, USA). A Magnesium Content Assay kit and collagenase II were purchased from Beijing Solarbio Science & Technology Co., Ltd. (Beijing, China). All reagents were used directly without further purification.

### 2.2. Preparation of Au Seeds

The Au seeds were synthesized using a simple method. CTAC (0.16 g) was dissolved in 10 mL of deionized water to prepare the CTAC solution [34]. Next, 50 μL of HAuCl_4_·3H_2_O (20 mg/mL) and 500 μL of sodium citrate (0.1 mol) were added to the CTAC solution. Then, 0.25 mL of AA (25 mmol) was also added to the CTAC solution and heated at 80 °C for 90 min to obtain the gold seeds (Au seeds).

### 2.3. Preparation of Au BNPs

A total of 100 μL of AgNO_3_ (0.1 mol) solution was dissolved in 100 mL of CTAB solution (100 mmol), and then, 2 mL of HAuCl_4_·3H_2_O (20 mg/mL) and 2 mL of hydrochloric acid (1 mol) were added [35]. Next, 1.6 mL of AA (0.1 mol) was added dropwise to form the growth solution. Then, 250 μL of the Au seeds were introduced to the growth solution and kept at 30 °C for 120 min. Finally, Au bipyramid nanoparticles (Au BNPs) were generated via centrifugation (5000 RPM for 20 min) and dispersed in 50 mL water. 

### 2.4. Preparation of Au@Pt BNPs

A total of 60 μL of H_2_PtCl_6_·6H_2_O (20 mg/mL) was added to the Au BNP (20 mL) solution. When the temperature reached 80 °C, 1.6 mL of AA (10 mmol) was added and the temperature was maintained for 30 min to obtain Au@Pt BNPs.

### 2.5. Preparation of Au@Pt@MgSiO_3_

First, Au@Pt BNPs (5 mL), TEOS (0.5 mL), and ammonia (0.46 mL) were added to ethanol (48 mL), and then stirred for 3 h and centrifugated (5000 RPM for 8 min) to produce Au@Pt@SiO_2_ (APS) nanoparticles [36]. Then, a solution (30 mL) was prepared, containing magnesium chloride (MgCl_2_) (0.8 mmol), ammonium chloride (10 mmol), and ammonia (1 mL). The APS NPs (1 mL) were dispersed into the solution and placed at 120 °C for 1 h to obtain Au@Pt@MgSiO_3_ nanoparticles.

### 2.6. Preparation of Thermal-Responsive Hydrogel

A total of 400 μL of APM NPs (1 mg/mL) was mixed with 400 μL of GelMA (10%); then, we added 50 μL of LAP (6%) and 1 μL of GM (2 mg/mL). Finally, the GAG hydrogel was formed via irradiation at 365 nm for 10 min.

### 2.7. Photothermal Performance of APM NPs

To study the photothermal effect, temperature changes in the APM NP solution were measured under 1064 nm laser irradiation. A thermal imaging system was used to detect the real-time temperature and to obtain thermal images of the APM NP solution at different concentrations (0, 100, 200, 300, and 400 μg/mL) and intensities (0.2, 0.4, 0.6, 0.8, 1.0, 1.5, and 2.0 W/cm^2^). In addition, the photostability of the APM NP solution was evaluated under 1064 nm laser irradiation over 5 cycles of an on/off process by monitoring the thermal imaging system.

### 2.8. Ability of APM NPs to Remove H_2_O_2_

To study the effect of APM NPs on removing H_2_O_2_, a portable dissolved oxygen meter was used for detecting the concentration of oxygen to indirectly reflect the removal of H_2_O_2_. A total of 10 mL of H_2_O_2_ solution (10 mmol) was prepared, and then, different concentrations of APM NPs (0, 2, 5, 15, and 20 μg/mL) were added. Finally, the change in oxygen concentration in the solution within 30 min was measured.

### 2.9. Ability to Release Magnesium Ions

To evaluate the Mg^2+^ generation capacity of APM NPs, Mg^2+^ detection kits were used for detecting the production of magnesium ions. The APM NPs were placed in PBS at different pH values, and we removed the PBS solution at different time points. Then, the absorption intensity of the PBS solution was measured to evaluate the release of magnesium ions by APM NPs at different pH values.

### 2.10. Degradation of GAG Hydrogels

To study the degradation of GAG hydrogels, the environment at the wound site was simulated by using collagenase type II. The GAG hydrogels were divided into three groups according to the proportion of GelMA and APM NPs: 100, 200, and 300%. Subsequently, 200 μL of hydrogel was placed in 0.5 mL of collagenase type II solution (2 U/mL). The solution was placed at 37 °C and treated at 80 rpm. The hydrogel was removed at different times and weighed and photographed.

### 2.11. The Photothermal Performance of GAG Hydrogels

We synthesized different hydrogels and investigated their photothermal ability. The hydrogels of the GelMA hydrogel (G hyd) and GelMA-GM hydrogel (GG hyd) groups were both synthesized without APM NPs. The hydrogels of the GelMA-APM hydrogel (GA hyd) and GAG hyd groups were synthesized with APM NPs. Consequently, the temperature change in the hydrogel under 1064 nm irradiation was measured.

### 2.12. The Release of APM NPs and GM

We used FITC small molecules instead of GM for the drug-releasing experiments. The hydrogel was placed in PBS, and then the PBS was removed after different durations of laser irradiation. Consequently, the changes in the APM NP absorption peak and FTIC fluorescence peak in the PBS solution were measured.

### 2.13. Bacterial Culture

All bacteria were first cultivated overnight on Luria–Bertani (LB) agar plates at 37 °C in a bacteriological incubator. After reaching the exponential growth phase, the MRSA cells were harvested via centrifugation and resuspended in a saline solution. The bacteria were adjusted to a 0.5 McFarland standard (1.5 × 10^8^ colony-forming units [CFU]/mL) and then diluted to a concentration of 1.0 × 10^8^ CFU/mL in Mueller–Hinton broth (MHB).

### 2.14. In Vitro Antibacterial Activity of GAG Hydrogels

Colony-counting analysis was performed by culturing MRSA strains on LB agar plates to determine the number of bacterial colonies. Briefly, the MRSA strains were grown on LB agar plates for 24 h at 37 °C. A 0.5 McFarland inoculum of MRSA was prepared in saline solution (0.9%) from the bacterial cultures. The bacterial suspension was diluted to a concentration of 1.0 × 10^8^ CFU/mL, and then 1 mL of the diluted bacterial suspension was added to each group for further experiments. These treatments were divided into five groups, including the control, GA Hyd, GAG Hyd, GA Hyd + NIR, and GAG Hyd + NIR groups. After NIR (1064 nm, 1.5 W/cm^2^) irradiation for 10 min, 100 mL of dilution of each group was spread on LB agar plates and incubated at 37 °C for 24 h. Finally, the number of bacterial colonies formed was counted to determine the effectiveness of the treatment.

### 2.15. Determination of Live/Dead Ratio

The MRSA suspension (1.0 × 10^8^ CFU/mL) was seeded in hydrogel in preparation for the experimental treatments. Afterward, the treated bacterial suspension was removed and washed with saline solution. SYTO 9 was diluted in sterile water and used at a final concentration of 10 mmol, and propidium iodide (PI) was diluted in sterile water and used at a final concentration of 5 mmol. The mixture of SYTO 9/PI dye and the bacterial suspension was incubated under dark conditions for 10 min, and then live/dead cells were observed using a fluorescence microscope.

### 2.16. The Cytotoxicity of APM NPs

A CCK8 assay was conducted to evaluate the cytotoxicity of APM NPs on L929 cells. The L929 cells were seeded in 96-well microplates with 1 × 10^4^ cells per well. After incubation for 24 h, the culture medium was replaced with fresh medium which contained different concentrations of APM NPs (0, 50, 100, 150, and 200 μg/mL). After incubation for 24 h, the activity of the L929 cells was detected using a CCK8 kit.

### 2.17. Cell Migration

In vitro, a scratch wound-healing assay was used to reveal the ability of Mg^2+^ to induce cell migration and proliferation. L929 cells were seeded in 6-well microplates with 3 × 10^5^ cells per well. After incubation for 24 h, a scratch was made on the bottom, and the culture medium was replaced with APM NPs. After incubation for 24 h, the cell migration before and after incubation for 24 h was studied with an inverted microscope.

### 2.18. In Vivo Wound Healing

All experiments involving animals complied with the Institutional Animal Care and Use Committee and the Administrative Committee of Laboratory Animals of Fujian Medical University (FJMU 2023–Y–1141). Balb/c mice (15–20 g) were provided by Shanghai SLAC Laboratory Animal Co., Ltd. The full-thickness circular wound models (d = 8 mm) were established on the backs of the mice. Each wound was infected with 30 μL of MRSA suspension with a concentration of 1.5 × 10^8^ CFU/mL for 24 h. After grouping the wounds (control, GA Hyd, GAG Hyd, GA Hyd + NIR, and GAG Hyd + NIR), the hydrogel was placed on the surface of the wound and irradiated at 1064 nm (0.5 W/cm^2^, 10 min). On days 1, 4, 7, and 10, the wounds were photographed and analyzed.

### 2.19. Statistical Analysis

All results are expressed as the mean ± standard deviation (SD). Statistical significance was calculated using a one-way analysis of variance (ANOVA) (*, *p* < 0.05; **, *p* < 0.01; ***, *p* < 0.001).

## 3. Results and Discussion

### 3.1. Synthesis and Characterization of APM NPs

**Figure 1:**
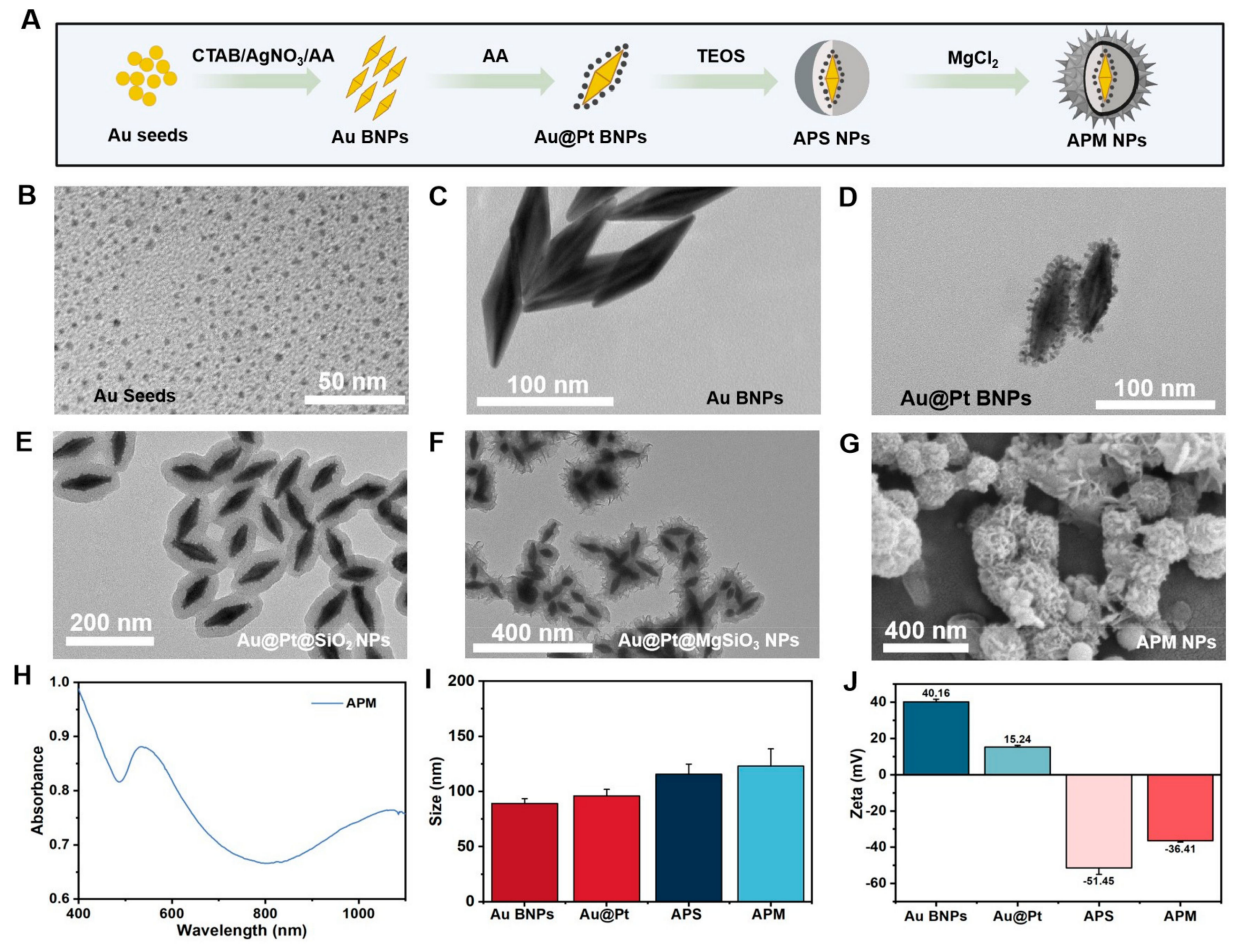
(**A**) Illustration of APM NP fabrication. TEM images of (**B**) Au seeds (scale bar: 50 nm), (**C**) Au BNPs (scale bar: 100 nm), (**D**) Au@Pt BNPs (scale bar: 100 nm), (**E**) APS NPs (scale bar: 200 nm), and (**F**) APM NPs (scale bar: 400 nm). (**G**) SEM image of APM NPs (scale bar: 400 nm). (**H**) UV−vis−NIR absorption spectra of APM NPs. (**I**) Size data and (**J**) Zeta potential of Au BNPs, Au@Pt BNPs, APS NPs, and APM NPs.

The APM NPs were prepared using a simple and convenient strategy (Figure 1A). First, ultra-small gold seeds (Au seeds) were successfully synthesized, which played an extremely important role in the formation of Au BNPs. Transmission electron microscopy (TEM) (Tecnai G2, FEI Company, Lausanne, Switzerland) data showed that the Au seeds had good dispersion, with a size of about 5 nm (Figure 1B). Then, Au BNPs were synthesized through seed-mediated growth under the action of AA and AgNO_3_. As shown in Figure 1C, the Au BNPs were characterized by long sides and a short middle. In order to decrease H_2_O_2_ levels, CAT-like Pt NPs were introduced to the surface of Au BNPs to form Au@Pt BNPs. As shown in Figure 1D, the Pt NPs were mainly distributed around Au BNPs, with a size of about 5 nm. To further promote the release of Mg^2+^, a magnesium silicate shell was synthesized via a hydrothermal reaction [37]. Through continuous agitation, a silicon shell with a thickness of about 20 nm was formed on the Au@Pt BNPs’ surface (Figure 1E). Then, the silicon shell was transformed into a magnesium silicate shell via a hydrothermal reaction (Figure 1F). These TEM images prove that the APM NPs were successfully synthesized through a series of processes.

The image obtained using the scanning electron microscope (SEM) (SU8100, HITACHI) further revealed that APM NPs had a uniform rough surface structure with a MgSiO_3_ shell, validating the success of rough-surface APM NPs (Figure 1G). Then, the absorption spectrum of APM NPs was studied, which showed prominent absorption peaks at 1064 nm (Figure 1H). This suggests that it is possible for APM NPs to act as PTAs in PTT-based antibacterial therapy. Moreover, the size distribution data revealed that the diameter of APM NPs was about 123 nm (Figure 1I), and the polymer dispersity index (PDI) (Malvern Zetasizer Nano ZS, Malvern, UK) was about 0.303. Meanwhile, the Zeta potentials of Au BNPs was 40.16 mV, of Au@Pt BNPs was 15.24 mV, of APS NPs was −51.45 mV, and of APM NPs was −36.41 mV (Figure 1J). These results collectively indicate the successful synthesis and characteristics of APM NPs, and their potential for use in PTT-based antibacterial therapy and remodeling of the wound microenvironment.

### 3.2. Property Analysis of APM NPs

#### 3.2.1. The Photothermal Performance of APM NPs

**Figure 2:**
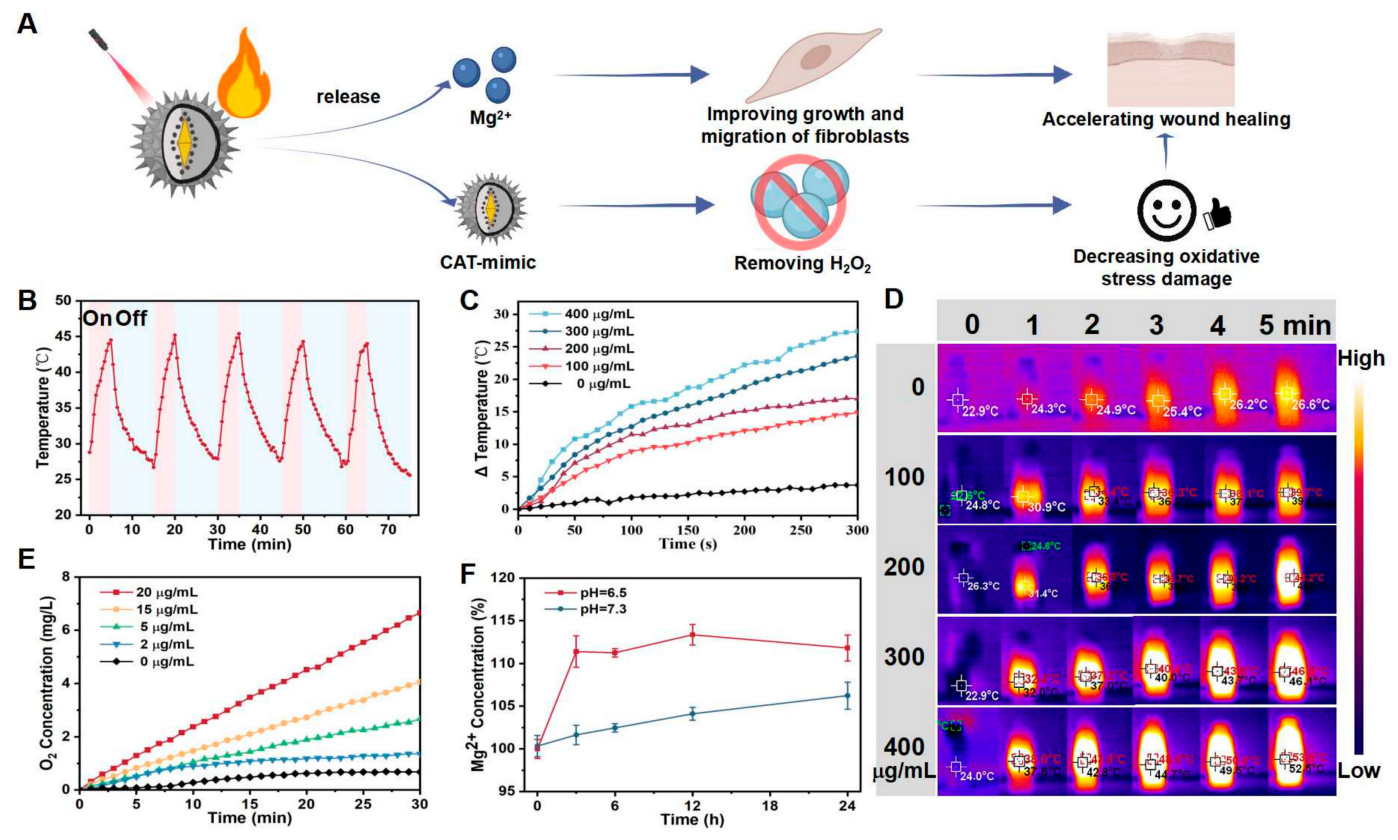
(**A**) Illustration of APM NPs to perform PTT, decrease H_2_O_2_, and release Mg^2+^. (**B**) Repeated heating experiments using APM NP solution over 5 cycles. (**C**) Temperature changes and (**D**) temperature photos of APM NP solution at different concentrations (0, 100, 200, 300, and 400 μg/mL). (**E**) Changes in O_2_ levels at different concentrations of APM NPs (0, 2, 5, 15, and 20 μg/mL). (**F**) Mg^2+^ release of APM NPs at different pH levels (6.5 and 7.5).

In order to evaluate the photothermal effect, temperature changes in the APM NPs solution at different parameters of 1064 nm laser irradiation were tested (Figure 2A). After five on/off cycles with 1064 nm laser irradiation, the APM NPs still exhibited photothermal abilities, indicating their excellent photothermal stability (Figure 2B). In order to further study the effect of APM NP concentrations on photothermal performance, the temperature changes were tested under NIR laser irradiation with a power density of 1.0 W/cm^2^, and temperature rise curves and thermal images were obtained (Figure 2C,D). With 0 μg/mL of APM NPs under 1064 nm irradiation, the temperature increased by only 3.7 °C. Moreover, with 400 μg/mL of APM NPs, the temperature increment was about 27.4 °C, confirming the light-to-heat ability of AMP NPs. Moreover, photothermal durability is also an important characteristic of APM NPs in PTT-based antibacterial therapy. Figure S1 shows that the temperature rise was more obvious in the APM group under 1064 nm irradiation after 15 min. In addition, the photothermal ability of APM NPs was studied in relation to laser intensity (Figure S2). It was found that the APM NPs had a considerable photothermal effect under 1064 nm irradiation, and can therefore be used for PTT-based antibacterial therapy.

#### 3.2.2. The CAT-like Performance of APM NPs

Due to the introduction of Pt NPs, we found that the APM NPs might have a CAT-like ability to catalyze the decomposition of H_2_O_2_ to produce O_2_. The decrease in H_2_O_2_ levels helped to decrease oxidative stress in the wound area. To study the CAT-like ability of APM NPs, we evaluated the oxygen concentration when mixed with different concentrations of APM NPs. As shown in Figure 2E, the O_2_ concentration in the control group (without APM NPs) changed slightly over time. When 20 μg/mL of APM NPs was added, the O_2_ concentration in the solution increased by about 6.7 mg/L, showing that the CAT-like APM NPs could decompose H_2_O_2_. Furthermore, Figure S3 shows that more and more oxygen bubbles could be observed on the pipe wall with an increase in APM NP concentration, verifying the CAT-like ability of APM NPs.

#### 3.2.3. Mg^2+^ Release in Acidic Environment

Due to the function of Mg^2+^ in promoting fibroblast growth and migration, we expected the designed nanomedicine APM NPs to be able to carry and release Mg^2+^. To verify this mechanism, a blood magnesium content assay kit was used to detect Mg^2+^ release. We compared the Mg^2+^ content between PBS solutions with different pH levels. When the pH was 7.3, the content of Mg^2+^ was slightly increased in the PBS solution, indicating that APM NPs may not produce many magnesium ions in a neutral environment (Figure 2F). However, when the pH dropped to 6.5, the level of Mg^2+^ in the PBS solution was significantly increased, which explains the Mg^2+^ release from APM NPs under acidic conditions. Therefore, APM NPs may release Mg^2+^ in an acidic environment, suggesting their potential in promoting wound healing.

### 3.3. The Performance of GAG Hydrogels

#### 3.3.1. The Synthesis and Characterization of GAG Hydrogels

**Figure 3:**
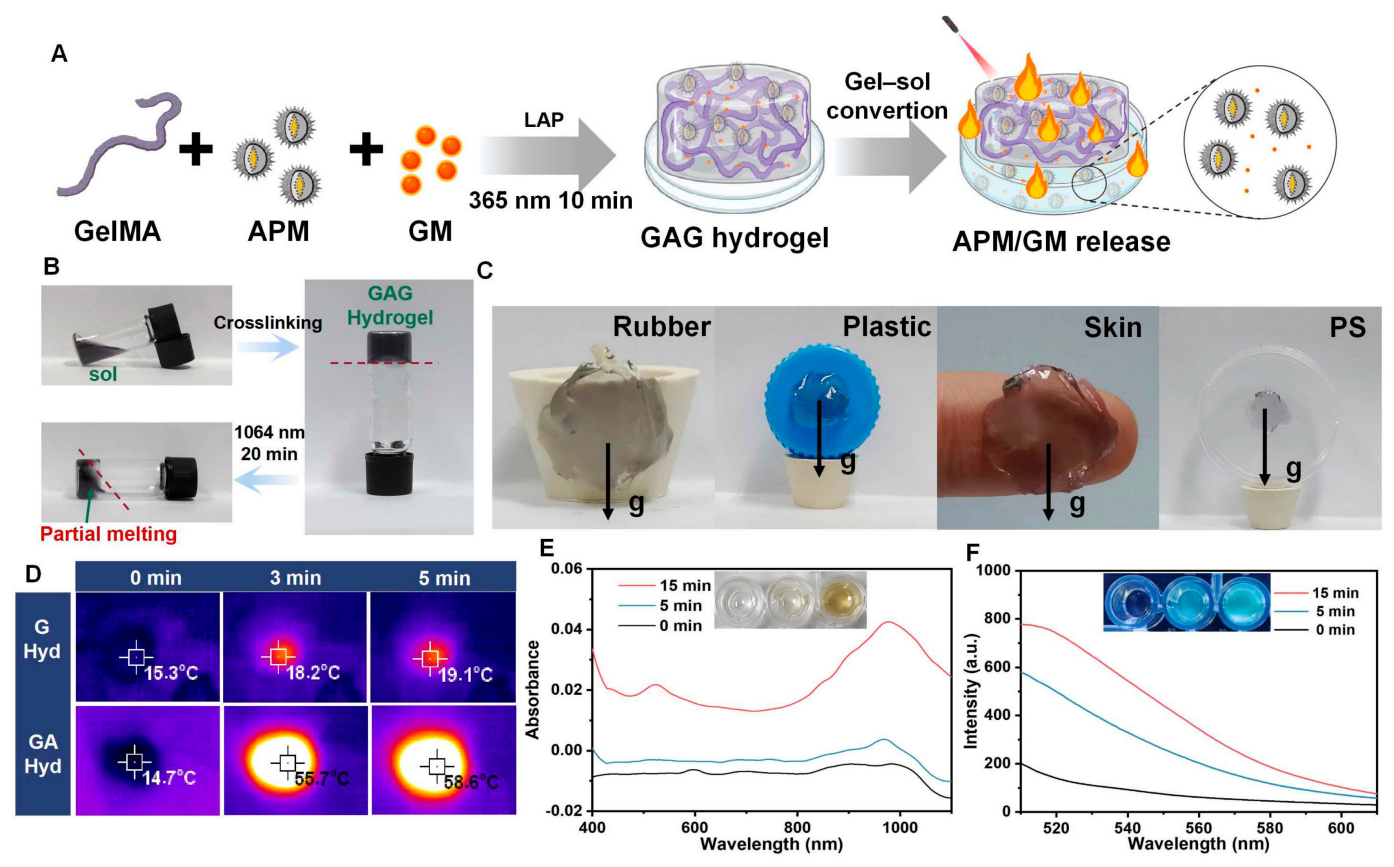
(**A**) Illustration of GAG hydrogel fabrication. (**B**) Photos of thermal-responsive GAG hydrogel in the process of synthesis and partial dissolution. (**C**) Photos of GAG hydrogels placed on the surface of different materials (rubber, skin, plastic, and PS). (**D**) Temperature photos of different hydrogels (G Hyd and GA Hyd). (**E**) The release of APM NPs at different durations of 1064 nm irradiation (0, 5, and 15 min). (**F**) The release of FITC (GM mimic) at different durations of 1064 nm irradiation (0, 5, and 15 min).

A thermal-responsive GAG hydrogel was prepared through LAP via a photo-crosslinking reaction (Figure 3A). The hydrogel appeared as a liquid and possessed fluidity before the photo-crosslinking reaction (Figure 3B). Then, after the photo-crosslinking reaction, transformation to a gel state occurred, indicating the successful synthesis of the GAG hydrogel. Subsequently, the GAG hydrogel was irradiated under a 1064 nm laser for 20 min, following which the hydrogel was partially dissolved. To explore the adhesion of the GAG hydrogel, it was placed on the surface of different materials and was found to adhere to them (Figure 3C). This showed that the GAG hydrogel had a certain adhesion ability, indicating its potential for filling in areas of skin loss. In addition, this soluble part returned to a gel state over time, which allowed the GAG hydrogel to more firmly adhere to the material (Figures S4–S6). These experimental results showed that the special thermal response capacity of GAG hydrogels might enable them to stick firmly to wounds, promoting wound healing. These experiments demonstrated the successful synthesis and the characteristics of thermal-responsive GAG hydrogels.

#### 3.3.2. Biodegradation of GAG Hydrogels

In order to verify the biodegradation, the GAG hydrogels were evaluated in PBS with collagenase II at 37 °C. As shown in Figure S7, the degradation ability of GAG hydrogels in different proportions was studied. The results showed that the GAG hydrogel with a proportion of 100% was completely degraded after 3 h of incubation in the pathological microenvironment, confirming the self-degradation properties of GAG hydrogels. This could prevent secondary damage caused by GAG hydrogels to the wound area. In addition, upon comparing different proportions of GAG hydrogels, it was found that the degradation rate was related to the GelMA content in the hydrogels. These experimental results provide a basis for studying the biosafety of GAG hydrogels.

#### 3.3.3. Photothermal Performance of GAG Hydrogels

Due to the inclusion of APM NPs, the GAG hydrogels were expected to have photothermal conversion properties. We evaluated the photothermal properties of GAG hydrogels under a 1064 nm laser irradiation. Figure 3D and Figure S8 show that the temperature of the GelMA hydrogel (G Hyd) without the addition of APM NPs exhibited little change and only increased by 3.8 °C after 5 min. After adding APM NPs, the temperature of the GA hydrogel (GA Hyd) increased rapidly by nearly 43.9 °C, indicating that the GAG hydrogel with APM NPs had excellent photothermal conversion ability. Moreover, the temperature changes in the other groups (the GelMA–GM and GAG hydrogel groups) are shown in Figure S9, indicating the photothermal ability of GAG hydrogels.

#### 3.3.4. APM NPs and GM Release of GAG Hydrogels

The GAG hydrogel exhibited a thermal-responsive performance, so it could be used for APM NPs and GM release. To verify the release of APM NPs, the UV–Vis–NIR absorption peak of the solution after laser irradiation was measured. The results in Figure 3E show that, with an increase in laser irradiation time, the absorption peak of the PBS solution increased significantly, indicating that APM NP release was constantly increasing. This showed that the APM NPs from the thermal-responsive GAG hydrogels could be released by adjusting the laser irradiation parameters. In addition, FITC fluorescent molecules were used instead of GM for hydrogel synthesis to explore the GM release property of GAG hydrogels. Figure 3F shows that the fluorescence signal of FITC increased with increasing laser irradiation time, indicating that GM in the GAG hydrogel could be released. Therefore, GAG hydrogels possess the capability of a thermal-responsive release of APM NPs and GM in the wound area.

### 3.4. Antibacterial and Microenvironment-Improving Activity of GAG Hydrogels

#### 3.4.1. Antibacterial Activity of GAG Hydrogels In Vitro

**Figure 4:**
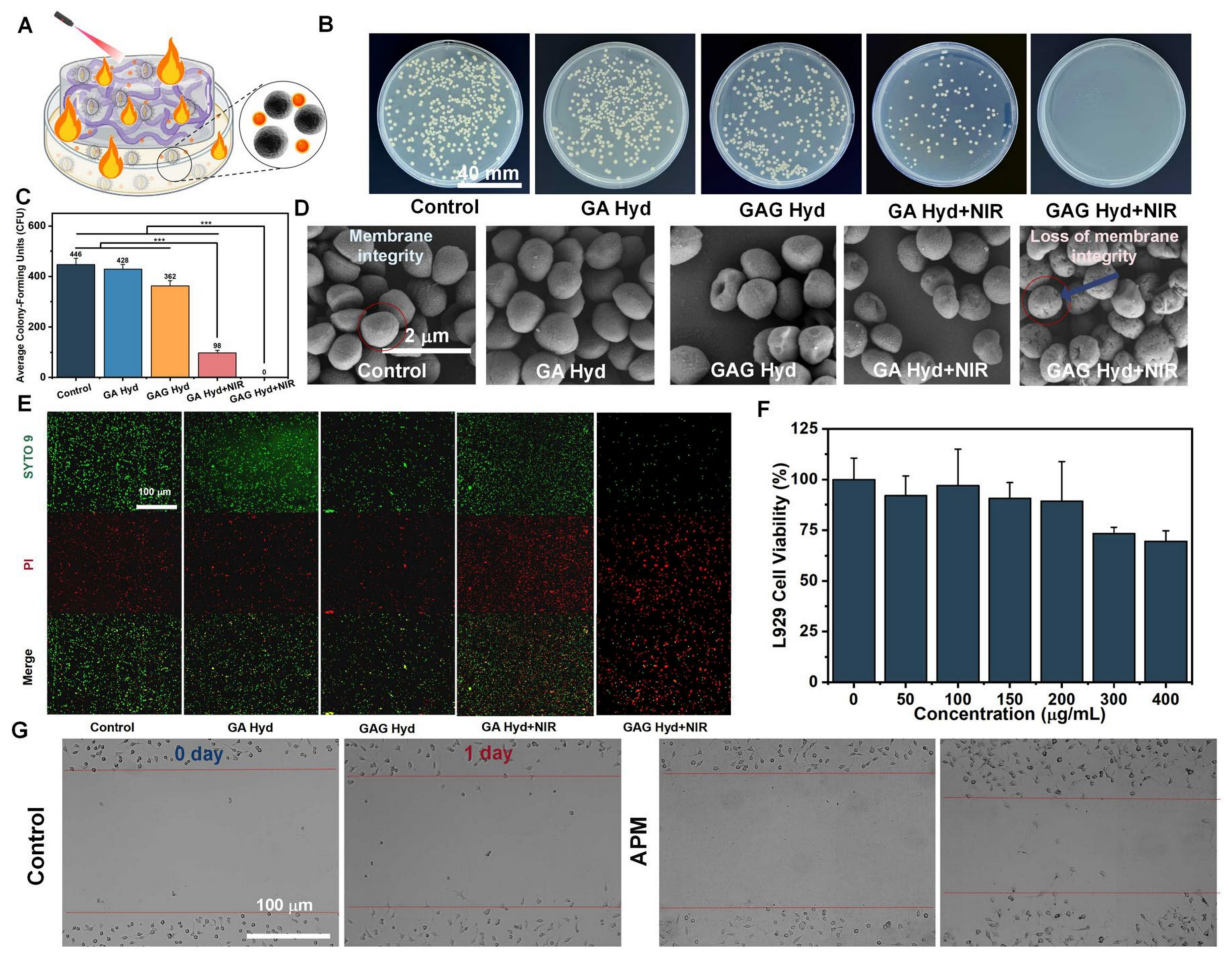
(**A**) Illustration of the GAG hydrogels used for antibacterial therapy in vitro. (**B**) Photographic images of MRSA colonies treated with different hydrogels (control, GA Hyd, GAG Hyd, GA Hyd + NIR, and GAG Hyd + NIR) (scale bar: 40 mm). (**C**) Average colony-forming units of MRSA in different hydrogels. (**D**) SEM images of MRSA treated with different hydrogels (control, GA Hyd, GAG Hyd, GA Hyd + NIR, and GAG Hyd + NIR) (scale bar: 2 μm). (**E**) Live/dead staining assay of MRSA in different hydrogels (control, GA Hyd, GAG Hyd, GA Hyd + NIR, and GAG Hyd + NIR) (scale bar: 100 μm). (**F**) Cytocompatibility of APM NPs at different concentrations (0, 50, 100, 150, 200, 300, and 400 μg/mL). (**G**) Cell migration of L929 by APM NPs (200 μg/mL) (scale bar: 100 μm), *** *p* < 0.001.

The GAG hydrogels were expected to induce hyperthermia and kill bacteria under 1064 nm irradiation (Figure 4A). To verify whether the GAG hydrogels possessed antibacterial abilities, bacterial experiments were performed, including a colony-forming assay, a bacterial morphology assay, and a live/dead staining assay. Based on the experimental results (Figure 4B,C), the average colony-forming units (CFU) of MRSA in different treatment groups were as follows: control group, 446 CFU; GA Hyd group, 428 CFU; GAG Hyd group, 362 CFU; GA Hyd + NIR group, 98 CFU; and GAG Hyd + US + NIR group, no bacterial growth. The colony-forming results showed that the control group and GA Hyd group without GM and NIR did not have an antibacterial effect. Upon adding GM (GAG Hyd group), the number of bacteria slightly decreased, while most bacteria still survived and the antibacterial effect was not obvious. In another group (GA Hyd + NIR group), the number of bacteria clearly decreased, indicating that PTT using the GA hydrogel had positive effects on antibacterial therapy. It is worth noting that the number of bacteria in the All group (GAG Hyd + NIR group) was 0 CFU, indicating that the GAG hydrogel had a strong antibacterial effect under 1064 nm irradiation.

The SEM images were used to observe the morphological changes in bacteria induced by different treatments. Figure 4D shows that the MRSA in the control group (without any treatment) and GA hydrogel group exhibited a nearly spherical shape with smooth surfaces. However, MRSA treated with the GAG hydrogel and GA hydrogel + NIR showed varying degrees of damage. The damage was particularly evident in the All group, with the bacterial surface showing wrinkling, indentations, and numerous pores, indicating the loss of membrane integrity. These results showed that the bacterial membrane underwent reduced integrity and increased permeability, which may accelerate the penetration of GM and the killing of bacteria.

To further assess the antibacterial ability of GAG hydrogels, an experiment using a combination of SYTO 9/PI dye and a bacterial suspension was conducted. The permeant nucleic acid dye SYTO 9 could enter all cells, while only membrane-damaged cells were stained red by the impermeant nucleic acid dye PI. Fluorescence images and the results of the quantitative analysis of MRSA targeted with different treatments are shown in Figure 4E and Figure S10. Only a few red spots were observed in the control group (without any treatment) and GA hydrogel group, while more red spots were observed for those treated with the GAG hydrogel and GA hydrogel + NIR. Additionally, the bacterial survival rates in the GAG hydrogel group and GA hydrogel + NIR group were 51.75% and 52.91%, respectively. Furthermore, the number of red spots increased significantly in the GAG hydrogel + NIR group, and the bacterial survival rate was only 16.5%, confirming the antibacterial effect of using GAG hydrogels in combination with PTT and GM release. These experimental results verified that the GAG hydrogel combining PTT and GM release possessed a good antibacterial ability.

#### 3.4.2. Cytotoxicity and Cell Migration Ability

To investigate the safety of APM NPs at the wound site, L929 cells were used to detect cytotoxicity. As described in Figure 4F, cell viability was about 100% in the control group (without APM NPs). Upon adding different concentrations (0–200 μg/mL) of APM NPs, cell viability was still greater than 80%, indicating that the APM NPs promoted biosafety for the L929 cells. Furthermore, an easy and affordable scratch test was used to evaluate L929 cell migration with APM NPs. The scratch gaps were evaluated at 0 h and 24 h to learn about the APM NPs’ promotion of cell migration ability (Figure 4G and Figure S11). At 0 h, there were very obvious scratch gaps in both the control group and APM NP group. At 24 h, the scratch gap in the control group showed almost no significant change, while the gap in the APM group was narrowed and the cells gradually migrated to the middle region. These results indicated that the APM NPs had good cytocompatibility and could increase cell migration to promote wound repair.

### 3.5. In Vivo Wound Healing Effects of GAG Hydrogels

**Figure 5:**
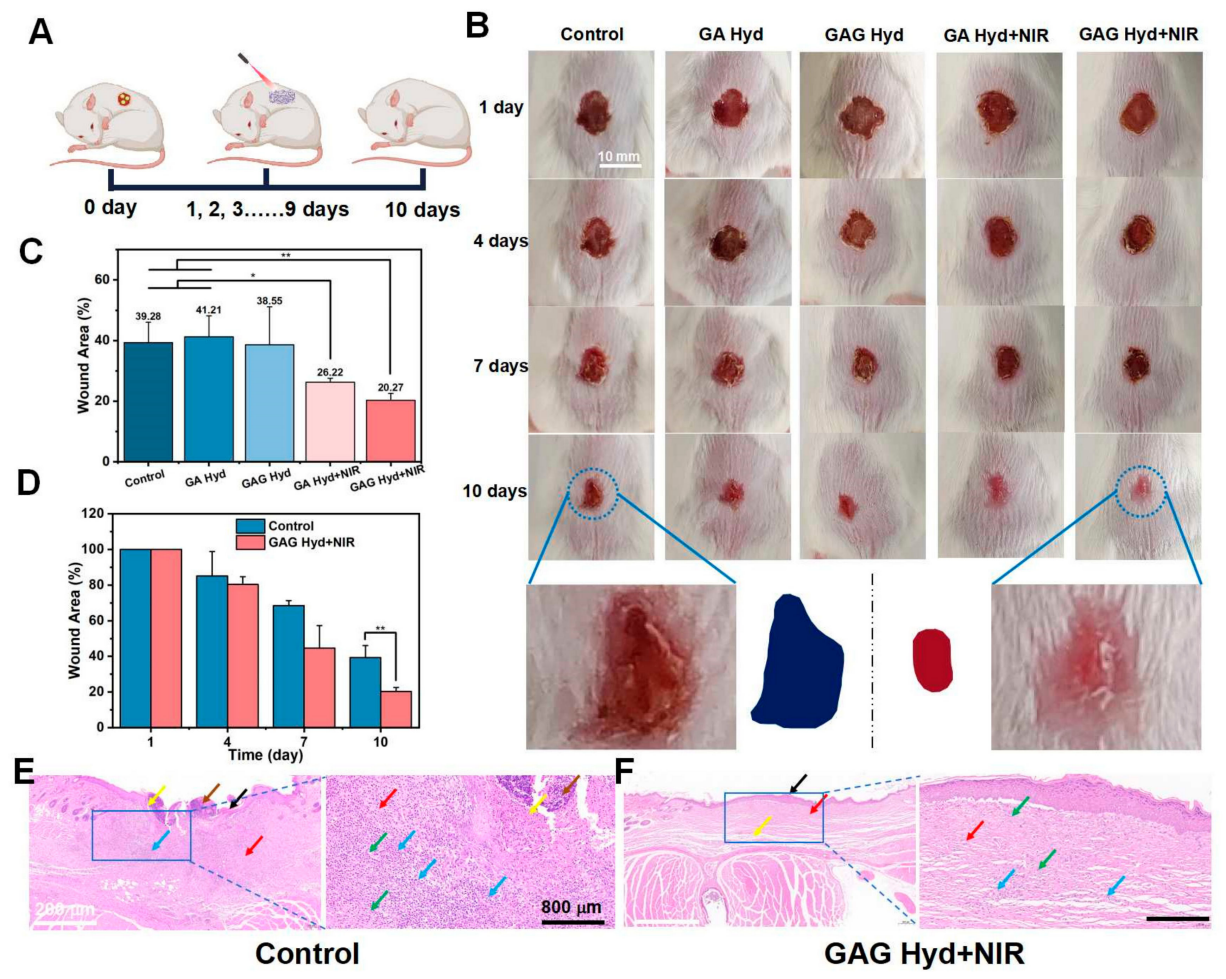
(**A**) Schedule of therapeutic strategies in vivo. (**B**) Photographs of mice after different treatments (control, GA Hyd, GAG Hyd, GA Hyd + NIR, and GAG Hyd + NIR) (scale bar: 10 mm). (**C**) Remaining wound areas following different therapies after 10 days. (**D**) Changes in remaining wound areas following different therapies (control and GAG hydrogel + NIR) over 10 days. (**E**) H&E staining evaluation of wound regeneration in control group (without any treatment) (scale bar: 800 μm and 200 μm). (**F**) H&E staining evaluation of wound regeneration with GAG hydrogel + NIR group treatment (1064 nm, 0.5 W/cm^2^, 10 min) (scale bar: 800 μm and 200 μm), * *p* < 0.05; ** *p* < 0.01.

These results showed that the thermal-responsive GAG hydrogels had the ability to kill bacteria, reduce antibiotics use, remodel the microenvironment, and promote cell proliferation, which can promote wound repair. Therefore, a full-thickness MRSA-infected wound model was selected to evaluate the wound repair ability of GAG hydrogels (Figure 5A and Figure S12). As described in Figure 5B,C, the wound in the control group without any treatment was still large after 10 days, and there was a scab on the surface. In the GA Hyd and GAG Hyd groups, the wound areas were similar to that in the control group, indicating that neither treatment had a therapeutic effect. The wound area of the GA Hyd + NIR group showed a significant reduction compared to the control group, which might be due to the killing of bacteria by PTT and the release of APM NPs to promote cell migration. In addition, the wound area was the smallest in the GAG Hyd + NIR group, and the wound healing effect was the most pronounced after 10 days. Furthermore, Figure 5D shows a comparison of wound area sizes over the 10 days with different treatment strategies, indicating that the thermal-responsive antibacterial GAG hydrogels combined with PTT and APM/GM release were highly effective in promoting wound healing. 

To further analyze the wound-healing ability of the different hydrogels, hematoxylin–eosin (H&E) staining was used to assess the process of wound healing. In the early stage of wound healing, inflammatory cells would be recruited, there would be an acute inflammatory response at the wound site, and the degree of inflammation would decrease with the progress of healing [2]. As shown in Figure 5E, in the control group, the epidermis was missing at the site of the skin tissue injury, and we noted an abscess (brown arrow), bleeding (yellow arrow), and a lot of necrotic cell debris. Additionally, the epidermis around the lesion was also thickened, accompanied by hyperkeratosis (black arrow) and stratum corneum thickening. Moreover, the dermis and subcutaneous structures were unclear, with no obvious skin appendages and a lot of connective tissue hyperplasia (red arrow). Furthermore, a large amount of lymphocyte and granulocyte infiltration (blue arrow) was shown. The results showed that there was an acute inflammatory response in the control group, indicating that the wounds healed slowly without treatment. After treatment with the GAG hydrogel under NIR irradiation, the epidermis was intact at the site of skin tissue injury (Figure 5F). We noted connective tissue proliferation (red arrow), neovascularization (green arrow), and reduced lymphocyte and granulocyte infiltration (blue arrow) at the wound site. These experimental results demonstrated that the thermal-responsive antibacterial GAG hydrogel possessed a good wound-healing capacity. Therefore, the thermal-responsive GAG hydrogel combining PTT and APM/GM release might represent a good therapeutic strategy for wound-healing therapy in the future.

## 4. Conclusions

In this study, we designed a thermal-responsive antibacterial GAG hydrogel for wound healing by loading CAT-like APM NPs and GM. The APM NPs were prepared using simple methods, including seed-mediated growth, a hydrothermal reaction, etc. The APM NPs were contained with Au BNPs, which exhibit good performance in photothermal conversion, reducing H_2_O_2_, and releasing Mg^2+^. Then, the GAG hydrogel was synthesized by mixing GelMA, APM NPs, and GM under a photo-crosslinking reaction. The GAG hydrogel possessed the ability to combine photothermal therapy and antibiotic therapy, which can kill bacteria, prevent antibiotics abuse, remodel the wound microenvironment, and promote cell proliferation. Under 1064 nm laser irradiation, the APM NPs in the GAG hydrogel generated local hyperthermia to kill MRSA, and the generated heat led to a change in GAG hydrogel morphology and the release of GM and APM NPs to prevent the overuse of antibiotics. Because of the enhanced bacterial cell membrane permeability caused by PTT, GM could more easily enter the bacteria and kill them. Subsequently, the CAT-like APM NPs decreased the H_2_O_2_ levels to reduce oxidative stress and regulate the wound microenvironment. Then, the APM NPs were decomposed to release Mg^2+^ under the weakly acidic wound microenvironment, promoting the growth and migration of cells for wound healing. Therefore, the proposed thermal-responsive antibacterial GAG hydrogel represents a promising solution in the field of wound healing.

## Data Availability

Data are contained within the article and Supplementary Materials.

## Supplementary Materials

The following supporting information can be downloaded at: https://www.mdpi.com/article/10.3390/antiox13070857/s1. Figure S1: (A) The temperature changes and (B) photo images in different solutions (water and APM NPs) during 15 min. Figure S2: The temperature changes in APM NPs solution at different laser intensities. Figure S3: The oxygen bubbles production in H_2_O_2_ solution with different APM NPs concentrations (0, 2, 5, 15, and 20 μg/mL). Figure S4: The adhesion changes before and after 1064 nm laser irradiation. Figure S5: The healing of GAG hydrogels after 1064 nm irradiation. Figure S6: (A) The hydrogel synthesized using 5 % of GelMA was unable to form a stable shape. (B) The hydrogel synthesized using 10 % of GelMA. The hydrogel forms a stable shape and has good adhesion to the skin. (C) The hydrogel synthesized using 20 % of GelMA. Hydrogels can form a stable shape, but the skin adhesion is not good. Figure S7: (A) The degradation of GAG hydrogels in different ratios (1:1, 2:1, and 3:1), and (B) the weight changes in GAG hydrogels. Figure S8: The temperature changes in different hydrogels under 1064 nm irradiation. Figure S9: Temperature photos of different hydrogels (GG Hyd and GAG Hyd). Figure S10: The analysis of bacterial survival rates after different treatments (Control, GA Hyd, GAG Hyd, GA Hyd + NIR, and GAG Hyd + NIR). Figure S11: The scratch test of L929 in different treatments (control, APM NPs, and MgCl2) after 1 day. Figure S12: (A) Changes in wound skin after NIR treatment in different groups (control and GAG hydrogel group). (B) Temperature change curves after different treatments (NIR and GAG hydrogel + NIR). (C) Temperature change after different treatments (NIR and GAG hydrogel + NIR).
