# Supplementary material for: Thermal-Responsive Antibacterial Hydrogel with Photothermal Therapy and Improving Wound Microenvironment for Promote Healing
Source: Antioxidants (Basel). 2024 Jul 17;13(7):857. doi: 10.3390/antiox13070857 (PMC11274332; doi:10.3390/antiox13070857)
Supplement: Supplementary file 1 [file antioxidants-13-00857-s001.zip › antioxidants-3064760-supplementary.pdf]

Thermal-Responsive Antibacterial Hydrogel with Photothermal Therapy and Improving Wound Microenvironment for Promote Healing

Linjie Huang [1,†], Jingwen Deng [2,†], Yina Su [1], Xueqi Hu [1], Yichao Zhang [1], Shanni Hong [1,*] and Xiahui Lin [1,*]

[1] School of Medical Imaging, Fujian Medical University, Fuzhou 350122, China
[2] Department of Otorhinolaryngology, Fujian Medical University Union Hospital, Fuzhou 350001, China
* Correspondence: snhong2020@fjmu.edu.cn (S.H.); xiahuilin@fjmu.edu.cn (X.L.)
† These authors contributed equally to this work.

Supplementary Materials

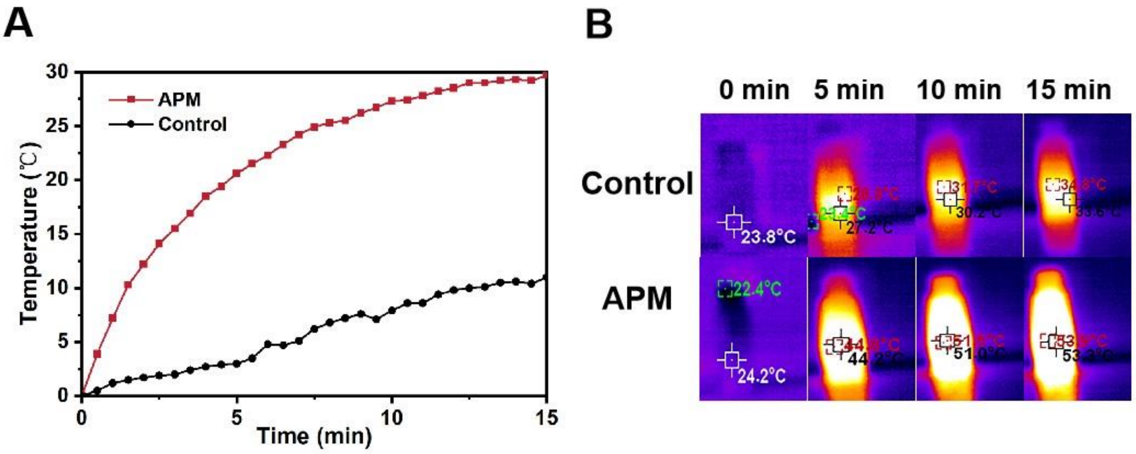

Figure S1. (**A**) The temperature changes and (**B**) photo images in different solutions (water and APM NPs) during 15 min.

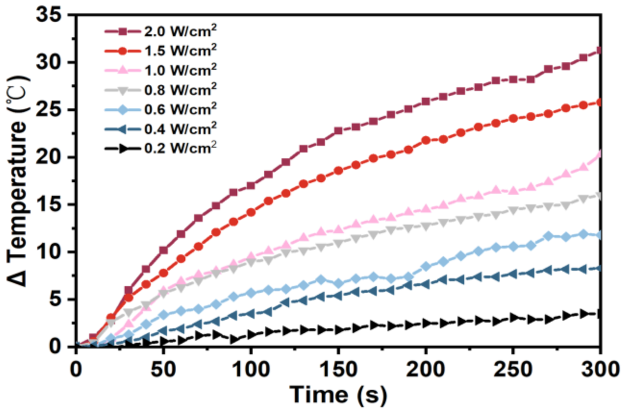

Figure S2. The temperature changes of APM NPs solution at different laser intensities.

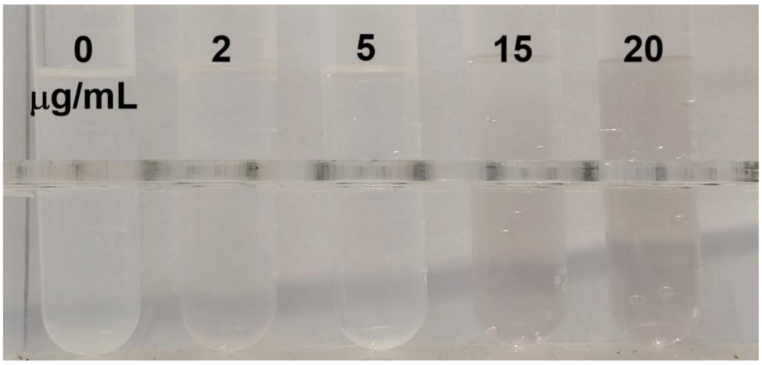

Figure S3. The oxygen bubbles production in H_2O_2 solution with different APM NPs concentrations (0, 2, 5, 15, and 20 mg/mL).

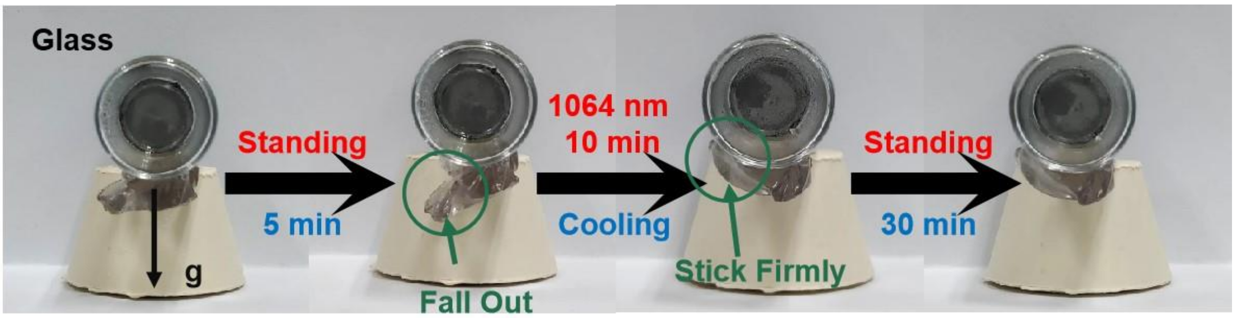

Figure S4. The adhesion changes before and after 1064 nm laser irradiation.

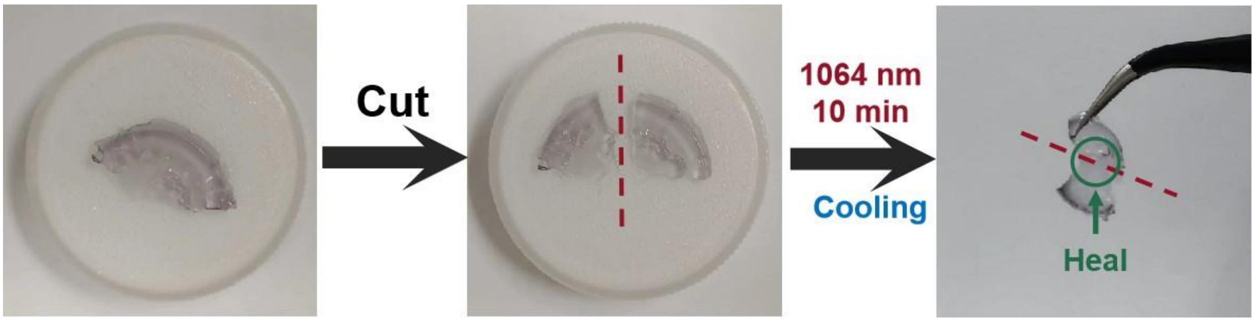

Figure S5. The healing of GAG hydrogels after 1064 nm irradiation.

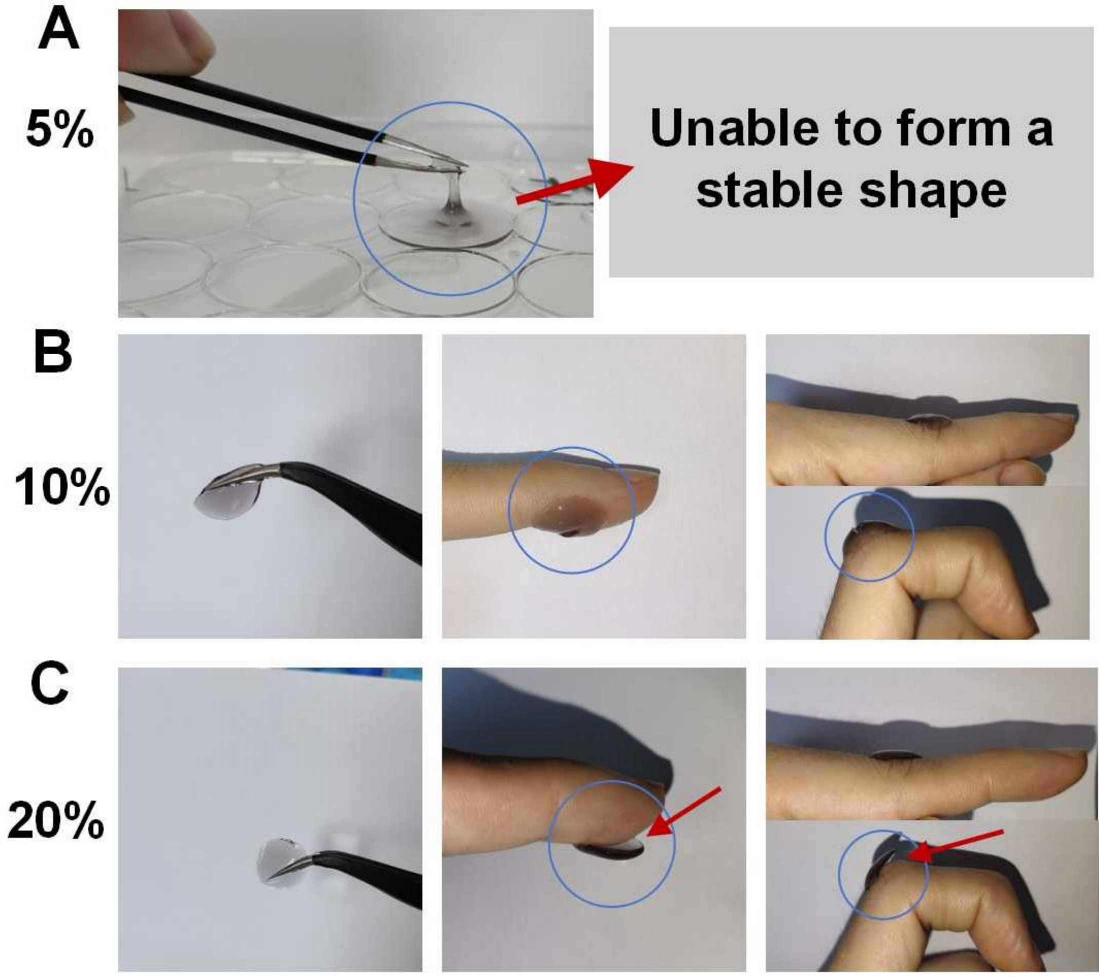

Figure S6. (**A**) The hydrogel synthesized using 5 % of GelMA. Unable to form a stable shape. (**B**) The hydrogel synthesized using 10 % of GelMA. The hydrogel forms a stable shape and has good adhesion to the skin. (**C**) The hydrogel synthesized using 20 % of GelMA. Hydrogels can form a stable shape, but the skin adhesion is not good.

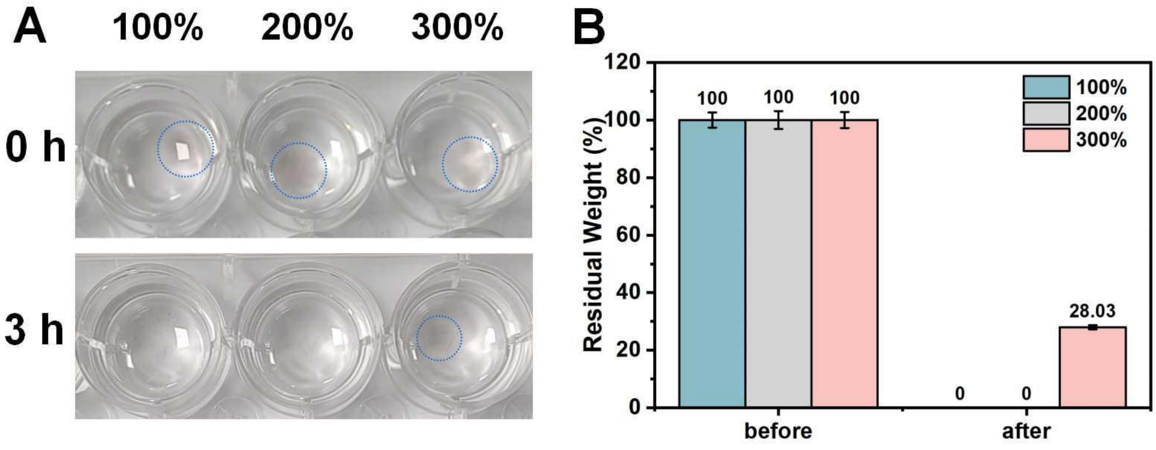

Figure S7. (**A**) The degradation of GAG hydrogels in different ratios (100, 200, and 300%), and (**B**) the weight changes of GAG hydrogels.

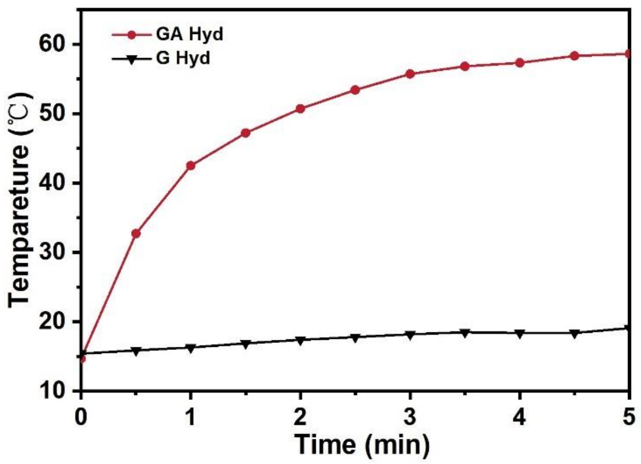

Figure S8. The temperature changes of different hydrogels under 1064 nm irradiation.

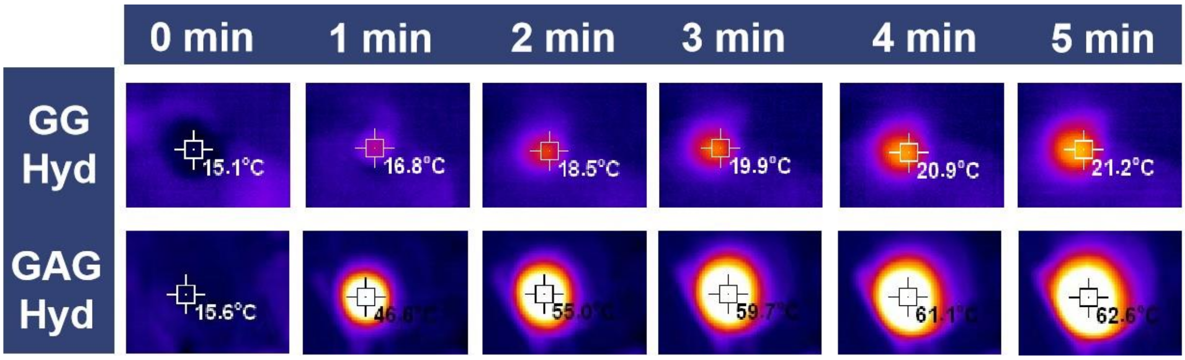

Figure S9. Temperature photos of different hydrogels (GG Hyd and GAG Hyd).

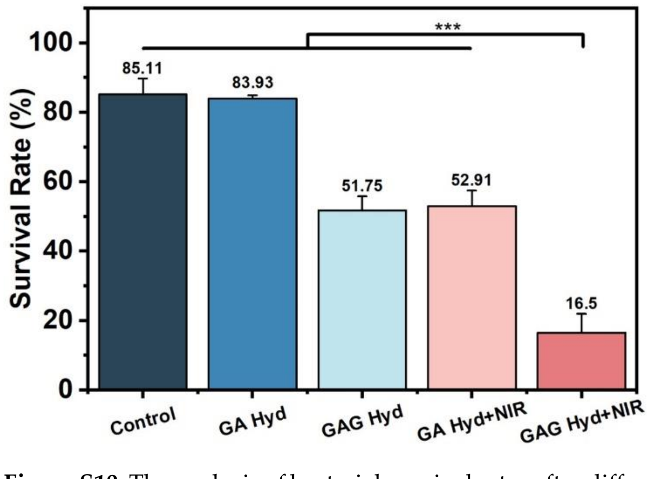

Figure S10. The analysis of bacterial survival rates after different treatments (Control, GA Hyd, GAg Hyd, GA Hyd+NIR, GAG Hyd+NIR).

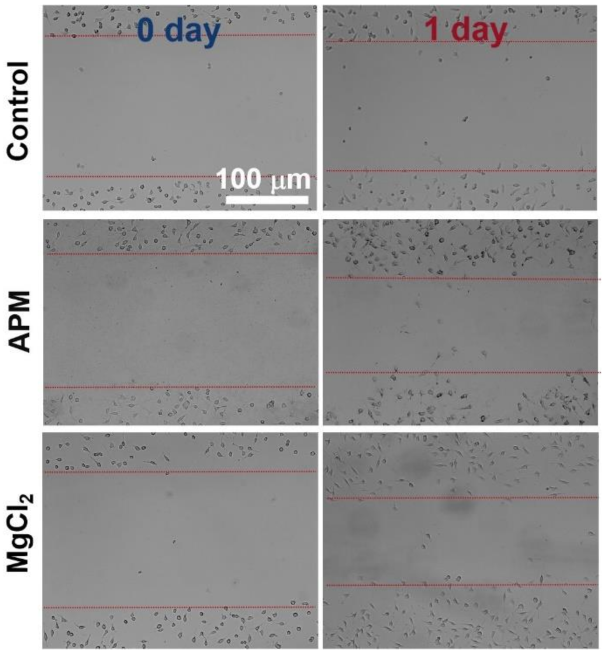

Figure S11. The scratch test of L929 in different treatments (control, APM NPs, and $MgCl_2$) after 1 day.

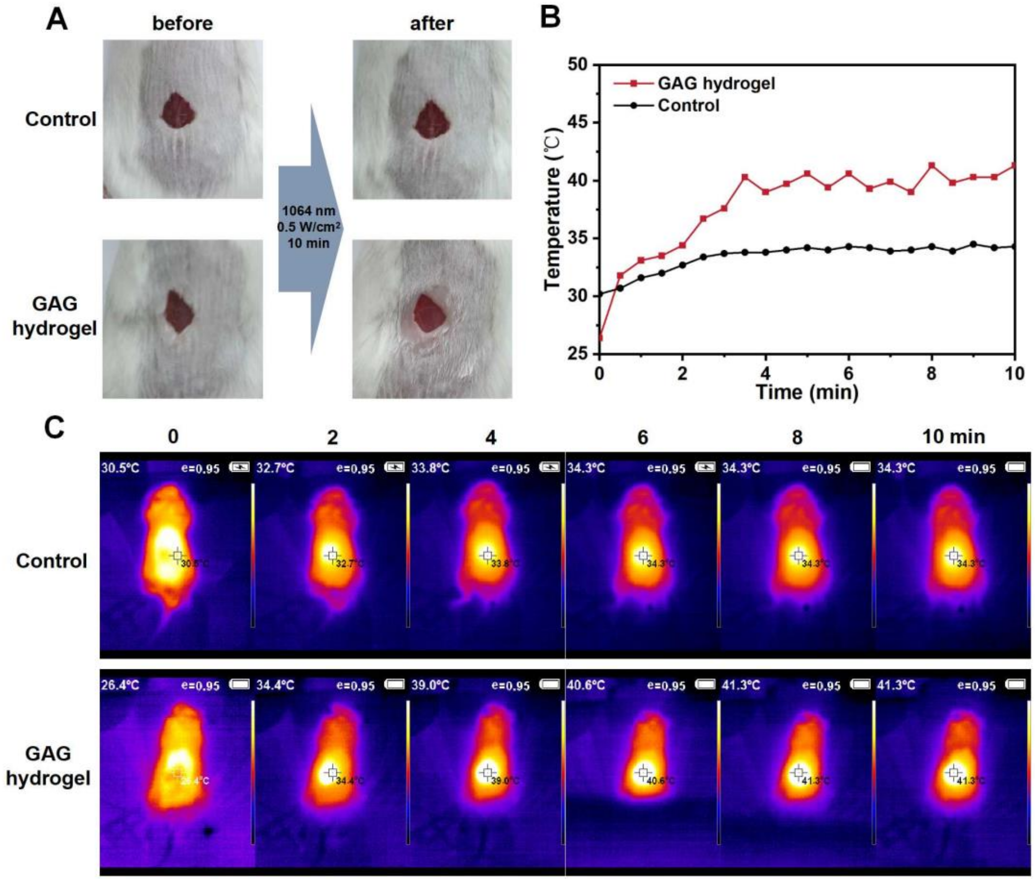

Figure S12. (**A**) Changes of wound skin after NIR treatment in different groups (control and GAG hydrogel group). (**B**) Temperature change curves after different treatments (NIR and GAG hydrogel+NIR). (**C**) Temperature change after different treatments (NIR and GAG hydrogel+NIR).
